# Postpartum opportunistic advice in primary care for women who have had gestational diabetes: a qualitative study of health care professionals’ views

**DOI:** 10.1186/s12875-021-01558-x

**Published:** 2021-10-20

**Authors:** Josie M. M. Evans, Aileen V. Ireland, Dawn M. Cameron, Kate M. Clarke, Claire E. Eades

**Affiliations:** 1 Faculty of Health Sciences and Sport, University of Stirling, Stirling, Scotland FK9 4LA UK; 2 Faculty of Social Sciences, University of Stirling, Stirling, Scotland FK9 4LA UK; 3 School of Health and Life Sciences, University of the West of Scotland, Glasgow, G72 OLH UK

## Abstract

**Background:**

Women who have had gestational diabetes during pregnancy are at very high risk of developing type 2 diabetes later in life, but their understanding of the risks is often limited. In this study we explored the views of health care professionals regarding offering brief opportunistic advice to women after their pregnancy, during unrelated consultations in primary care, relating to reducing diabetes risk.

**Methods:**

The study took place in three Health Boards in Scotland. We conducted semi-structured one-to-one interviews (either face-to-face or telephone) with two health visitors, three practice nurses, two GPs, two diabetes consultants and two obstetricians. A focus group with five health visitors was also held. A topic guide was followed, covering the feasibility and acceptability of delivering brief opportunistic advice during a routine consultation, the optimal way to identify and recall women with previous gestational diabetes, and the possible content and timing of any such intervention. A thematic approach was used to analyse the qualitative data generated.

**Results:**

The interviews/discussion lasted from 15 to 51 min. There was widespread support from all participants for offering opportunistic advice, and general consensus that health visitors would be best placed to do this as part of the Universal Health Visiting Pathway in Scotland. Thematic analysis generated three significant points of discussion: implications for training of health visitors, the need for a systematic approach to identifying women with gestational diabetes, and the optimal timing of delivery. Despite an already demanding schedule of providing advice and education to women, health visitors were confident that they could offer educational advice, provided that they received appropriate training to do so. However, there would need to be a watertight system for identifying women in their care who had had gestational diabetes. In terms of timing, later visits around 6–8 months after delivery were considered most suitable.

**Conclusions:**

There is support from health care professionals, and most pertinently from health visitors, that the frequency of routine visits with women during the Universal Health Visiting Pathway programme in Scotland provides potential opportunities for education around future diabetes risk to women who have had gestational diabetes.

**Supplementary Information:**

The online version contains supplementary material available at 10.1186/s12875-021-01558-x.

## Background

Over 5% of all pregnancies in Europe are estimated to be complicated by gestational diabetes mellitus (GDM) [1]. Although the overall prevalence is lower in Scotland, this figure is increasing [2]. Women who have had GDM during pregnancy are at very high risk of developing type 2 diabetes (T2D) later in life. Their risk of T2D is nearly 10-fold that of women who have not had GDM [3]. While reduced risk of T2D after GDM has been documented in women who have successfully made improvements to diet and physical activity levels post-delivery, effective interventions within heath care settings to achieve this are intensive and costly [4]. We have recently completed qualitative research that has identified that there are illness perceptions surrounding GDM that, if they are not addressed, are likely to compromise the effect of lifestyle interventions [5, 6]. For example, although many women know that there is an association between GDM and T2D, they may not perceive themselves personally as being at increased risk. For some women, GDM has minimal and transitory impact on their lives. If it is short-lived and easily controlled, they often do not appreciate the potential consequences. They may not regard T2D as particularly serious, and the lack of after-care postnatally, along with the limited mention of GDM or T2D by health care professionals, downplays its seriousness. This ‘postpartum abandonment’ has also been identified in other studies [7, 8].

In Scotland, the majority of women receive health care from the National Health Service (NHS), a government-funded health care system. Maternity care is free at the point of delivery. Women who are diagnosed with GDM during pregnancy are referred to a specialised diabetes clinic. They are also invited to a 90-min education session, usually led by midwives. Around half of these women manage their GDM with dietary change alone, while others may need to be treated with oral hypoglycaemic agents or insulin. Post-delivery, women are referred to their GP for an HbA1c test at 13 weeks (previously this was a 6-week check), although not all women attend. There are no standardised systems in place in primary care for continued monitoring of women who have had GDM, with different approaches to follow-up, depending on the GP practice.

There is growing interest in the use of brief opportunistic advice in the context of unrelated consultations in primary care to tackle obesity and prompt other behavioural changes [9]. For women with GDM, these could reduce the risk of GDM in future pregnancies, reduce their own risk of T2D and have far-reaching implications for the health and health behaviours of their offspring. Women who have given birth in Scotland have frequent health care consultations in primary care settings, many of them with health visitors. A health visitor is a qualified nurse (or midwife) with specialist training in child and family health. They complete a schedule of home visits to new mothers, known in Scotland as the Universal Health Visiting Pathway (HVP), to offer support and advice relating to the wellbeing of the child. There are 11 home visits (eight in the first year of life and three between 13 months and 3–5 years) [10]. Women may also have regular contact with practice nurses for cervical smear tests.

The objective of this study was to elicit the views of health care professionals (HCPs) as to whether health visitors or practice nurses could be used to deliver brief educational advice to women who have had GDM relating to behaviour change and diabetes risk. We conducted qualitative research in three Health Boards in Scotland, involving seven health visitors, two General Practitioners (GPs), three practice nurses, two consultant obstetricians and two diabetologists.

## Methods

In this study we used an opt-in convenience sampling approach among health visitors, practice nurses, GPs and consultants in three Health Boards in Scotland, with populations of 300–400,000. These included a geographically large rural Health Board (A), and two Health Boards in central Scotland, each with a large urban centre (B and C).

All health care professionals (HCPs) were approached by email, either from general staff lists or through personal contacts. They were informed of the purpose of the study (a Participant Information Leaflet was enclosed), and they were asked to contact the researcher if they wished to take part in an in-depth interview, or in a focus group discussion with other individuals. The researcher then followed up with telephone contact to make arrangements. There were no specific inclusion/exclusion criteria apart from that participants belonged to one of the HCP groups in one of the three Health Boards in the study. There was only one potential HCP, who showed an interest in taking part, with whom we were then unable to arrange a mutually convenient appointment.

Eleven interviews were carried out between February and April 2019, either by telephone or face-to-face with the postdoctoral researcher (AI), and one focus group was held with health visitors. The face-to-face interviews took place on either hospital or GP premises. The focus group took place in a local Council office and was facilitated by the researcher (AI), together with an academic nurse/midwife lecturer (DC). No other people were present, except for a student health visitor who was in attendance, observing the focus group. The post-doctoral researcher and the academic nurse/midwife were both female, and were experienced researched researchers, having completed PhD studies. DC was also clinically qualified and had previously worked as a midwife/ Health Visitor, while AI was new to the topic area. Any previous contact with participants was limited to brief professional interaction with DC, and participants were made aware of the interviewers’ backgrounds, and that this was a publicly funded study.

A topic guide was used for all discussions (interviews and focus groups), and was initially devised according to the RE-AIM framework [11]. Discussions then focused on three main topic areas: the feasibility and acceptability of delivering brief opportunistic advice during a routine consultation, the optimal way to identify and recall women with a previous GDM diagnosis, and the possible content and timing of any such intervention. This semi-structured format ensured that the topics of interest were covered while allowing participants the freedom to discuss any issues not covered in the guide. Interviews lasted between 15 and 51 min (median 23; IQR 10). Written informed consent was obtained from all participants.

Discussions were audio-recorded with the participants’ permission and transcribed verbatim by a professional transcription service (but not returned to the participants). A thematic approach to analysis was used to identify patterns and themes within the data and capture participants’ perceptions of offering brief opportunistic advice to women after pregnancy [12]. JE and AI repeatedly read the transcripts and collated relevant excerpts manually, which were analysed to reveal underlying meanings. AI generated initial codes, which were shared with the research team, then coded into sub-themes. These were reviewed against the data as a whole in an iterative and ongoing manner before over-arching themes were derived and named.

## Results

Eleven health care professionals (HCPs) were interviewed individually. This included two health visitors, two GPs, two diabetologists, two obstetricians and three practice nurses. Five health visitors took part in a focus group discussion (Table 1). All were female.

**Table 1:** Details of interviews/focus groups carried out

	Health Board A	Health Board B	Health Board C
Health Visitors	Focus group 5 participants (51 mins)	1 face to face interview: (24 mins)	1 telephone interview: (31 mins)
	p1 to p5	p6	p7
GPs	1 telephone (23 mins)	1 face-to-face interview (15 mins)	–
	p8	p9	
Obstetricians	–	2 face-to-face interviews (18,19 min)	–
		p10, p11	
Diabetologists	–	2 face-to-face interviews (16,23 min)	–
		p12, p13	
Practice Nurses	–	1 face-to-face interview, 1 telephone interview (20, 24 min)	1 telephone interview (28 min)
		p14, p15	p16

The interviews generated discussion surrounding women’s perceptions of GDM, the need for educational advice and the most appropriate HCP to deliver this advice. These findings are summarised in the section ‘Context’ below. Thematic analysis generated three important over-arching themes: implications for training of health visitors, the need for a systematic approach to identifying women with GDM, and the optimal timing of delivery of opportunistic advice.

### Context

There was unanimous agreement that an educational intervention for women with GDM in primary care was needed, with a typical view that ‘*it’s just so important, it makes so much sense*’ (p7). This was against a backdrop of the rise in the incidence of GDM, and the ‘*epidemic*’ of diabetes (p10), meaning that the ‘*service was being swamped*’ (p12). There was recognition that some women were unaware of their increased risk of T2D or thought that it did not apply directly to them, and that many women regarded GDM as a transient diagnosis, as evidenced by the following comments:

*I’m not even sure if they know they’re at increased risk of diagnosis of diabetes ... [the women say] ‘It’s happened when I was pregnant, it’s over now, pregnancy is over, I can go back to normal now’.* (p7).

*they’re not perceiving themselves to be at risk.* (p8).

This view was also endorsed by a diabetologist who was ‘*frustrated’* (p11) with the situation, but recognised that postnatally ‘*there is a good opportunity for health promotion’* (p10):

*And then they come back to you, 2 years on, probably heavier than they were the time before, and they’re older, they need more treatment, everything is worse. So although they seem to buy into it in pregnancy, nothing happens afterwards*. (p11).

Despite the recognised need for an intervention, a note of caution was raised by GPs and health visitors about opportunistically offering advice in primary care. Their concerns related to the ‘*psychological impact of a dialogue … about health and exercise’*, and the need to ensure  that this would not leave women with the over-riding sense of being ‘*told that they’re fat’* (p8)*.* One worried ‘*about lowering the woman’s self-esteem if we already know it’s low and then we start talking about her weight’* (p1) and how *‘it’s putting pressure on them’* (p1) at a challenging time. They also emphasised that the advice needed to be evidence-based.

Despite this, the central importance of behaviour change for both mother and her family was summed up well:

*So it’s trying to transfer the motivation that they have and the regular contact with health professionals they have during pregnancies after they’ve had the baby, so it’s trying to engage them with that, to have that few months of really engaging them to really think, promoting the fact that this isn’t just about this 9 months, this is about after you’ve had this baby and it’s for the next pregnancy and this is about 10 years down the line, this is about the impact on your whole family and further generations.* (p10).

Although two health visitors thought ‘*the GP is the best person to raise it’* (p1), common views from other HCPs were that that health visitors, rather than practice nurses, were best placed to deliver opportunistic advice, as ‘*they have more contact with the women and they visit them at home*’ (p14). One of the GPs agreed that ‘*health visitors are the ones who have got the most contact*’ (p9) and that contact with practice nurses might be too long after delivery to be effective. For this reason, the results presented below relate to views surrounding delivery of advice specifically by health visitors.

### Training

There was evident concern that health visitors (and other HCPs) might need ‘*particular knowledge’* (p13) and tailored education around GDM and its risks before they felt able to deliver educational advice, despite being potentially ‘*very capable*’ of doing so (p10). One health visitor remarked: ‘*I think I’ve had my awareness raised today’* (p4), with another responding ‘*it emphasises the need for appropriate training for the likes of us’* (p1). There was a sense that they would welcome more training in ‘*just knowing the questions to ask*’ (p4) and ‘*just knowing the facts’* (p1), and there was also the following admission:

*So you can understand mothers, and some professionals, maybe thinking that is a pregnancy-related condition* (p7).

One of the obstetricians also recalled that health visitors *‘felt they didn’t have the right information … the right training to try and deliver that kind of intervention*’(p12).

### Identification of women with GDM

In terms of the feasibility of delivering brief educational advice, there were no systematic or watertight systems by which health visitors could be sure they knew of all women in their care who had had GDM, and this uncertainty was clearly evident: ‘*You would know who was gestational diabetic wouldn’t you?*’ (p5). The uncertainty is further demonstrated in the following exchange:

*We’ve got one lady at the moment haven’t we … She’s very overweight. She didn’t have diabetes did she? (p2) She did (p3).*

The exception was the health visitor from Health Board C, who was more confident about this, despite receiving no ‘*formal notification*’ (p7). She referred to carrying out increasing numbers of ante-natal visits where this information would be passed on, either electronically or by the woman herself, or during the handover from the midwife. However, health visitors from the other two Health Boards indicated that the information was not always received from midwifes, given that this ‘*communication isn’t as good as it used to be when we were co-located with the midwives*’ (p1) and that the verbal handover from the midwife at discharge does ‘*not always*’ happen (p2). One health visitor commented, ‘*I’ve had many that have had it and when that’s not been passed on*’ (p3). However, other explanations for this were offered. For example, GDM may be diagnosed late on in pregnancy after any handover. The relevant information ‘*could potentially get a little bit missed in amongst everything if it’s not highlighted*’ (p6). Finally, there was a recognition that the main focus was the baby’s notes, which legally needed to be kept separate from the mother’s notes.

Health visitors continue to have contact with families until the child is 5 years old. Both GPs reported that a Read code is added to the medical records of women with GDM, and that this is used for recall for blood glucose tests after delivery. While they ‘*presumed*’ that health visitors were aware of GDM-complicated pregnancies, the systems ‘*would need to be a wee bit more joined up*’ (p9) for continued follow-up, and for Read codes to be used to identify older children (and associated health visitors’ visits) whose mothers had had GDM. This is because the health records of parents are not currently linked with those of children in the GP practice. The health visitor mostly receives information relating to the mother and baby from a hospital or community midwife. Overall, the proportion of mothers with GDM within one GP practice would be relatively low, so an efficient recall system would be needed. However, this could be relatively low-tech, for example, health visitors suggested having triggers (e.g. a sticker) on their paperwork, or GDM being added to the existing postnatal checklist. It was also suggested by one practice nurse that the reception team could flag up any women who missed their appointment for their postnatal GDM test (although this would not identify all women who had had GDM).

### Timing

The main challenge for health visitors to be trained and deliver educational advice was perceived to be their workload, given that ‘*these people, these professions are so hard-pushed as it is, they don’t have any more time to give or to work’* (p10). They already have a prescriptive list of topics that need to be covered at every visit as part of the universal pathway [9] for health visitors; there are always ‘*competing priorities*’ (p7), and it is also important that parents are not ‘*overloaded*’ (p7). There was widespread agreement that the 6-week visit would probably be too early for delivering behaviour change advice, as the visit is so ‘*focused on the baby or the child’* (p4), and mothers have ‘*so many other preoccupations with feeding the baby, lack of sleep, might have postnatal depression. Could be all sorts of things going on, so they’re very much focused on just surviving day to day often*’ (p1).

While one of the practice nurses suggested that the 12–14 week health visitor check was ‘*a nice time because that’s quite early on in the process*’ (p16), several health visitors mentioned the importance of the relationship that is built up with the mother, and that this kind of advice would be better delivered when ‘*you’ve got a bit of a relationship going and you hope they think you’re okay and everything*’ (p1). Also, in the later visits, things are often more ‘*relaxed*’ (p6) and by the 13–15 month visit ‘*women are more willing to engage about themselves rather than just their child*’ (p5). The optional visit at 6 months or the 8-month visit was deemed most appropriate for opportunistic advice, alongside advice about weaning and food preparation for the family, but there was widespread opposition to immunisation appointments being suitable:

*I wouldn’t think immunisations is a good point, because parents will be really stressed actually…* (p6).

## Discussion

In this study we were able to elicit the views of 16 health care professionals (HCPs) from three Health Boards in Scotland, around delivering brief educational advice to women with GDM in primary care after delivery. Although there may have been some variations in provision of care to women with GDM between the Health Boards, their views and experiences surrounding the topic seemed fairly consistent. They also chimed well with findings from other studies in a variety of settings, which suggest that many women with GDM, for various reasons, do not recognise the serious long-term implications of their GDM diagnosis [4–8]. Our findings respond to the recognition that a shift in perception of GDM is needed [13], from one where it is assumed to be a treatable short-term condition, to one that has the potential to influence the long-term health of mothers and their children in the future.

There was general consensus in our sample of HCPs that health visitors are well placed to offer opportunistic advice to women who have had GDM. Given that practice nurses have less contact with women and they also viewed health visitors as more suited to offering advice, the results we have presented relate mainly to health visitors. However, it is important to note that we used an opt-in convenience sampling for HCPs, so we cannot be sure how widespread the views of our participants were, nor whether those who were interviewed were those with particularly strong views or an interest in the topic. The nature of the funded study meant that we had a small sample size and were not able to formally assess data saturation, and that transcripts and results were not returned to the participants for comment. These are also acknowledged as study limitations. Despite this, we believe the following key points are worthy of consideration.

Some of the barriers to delivering opportunistic interventions raised in this study have also been identified by other HCPs in a range of contexts [13]. These include time and workload pressures, worries about harming the HCP-patient relationship by raising sensitive issues, insufficient training and concerns about capability [13]. However, ensuring that any sensitive issue can be raised and addressed is based on the key health visiting principle of forming trusting relationships with women [14]. Health visitors are skilled professionals educated to recognise and manage sensitive issues routinely in their practice. They are already required to screen for post-natal depression, and to raise the question of domestic abuse. They may often be preferred over other HCPs for discussing such personal issues; conversations can take place in the private spaces of women’s homes and where time pressures are less problematic [15]. Women often report being confident in the ongoing support and guidance they receive from their health visitor, with whom they have built a relationship. However, health visitors might require more tailored training before they feel confident in delivering opportunistic advice relating to diabetes risk. As the incidence of GDM increases, more HCPs without specialised training in diabetes will be involved de facto in the care of women who have had GDM, and while they may already discuss lifestyle issues of healthy diet, weight and exercise with families delivering key public health education messages from the HVP, they also need to be confident about diabetes-specific advice.

The need for a systematic, universal and effective approach for following up women with GDM has long been recognised [16]. GDM recall registers would be relatively straightforward to set up in primary care, and have been shown to be successful in other countries  [17, 18] but ensuring that health visitors are aware of women who would benefit from opportunistic advice around T2D risk is more problematic. There appears to be no national consistent approach to handover information from midwife to health visitor, each Health Board having local arrangements in place and midwives sharing information with the health visitor they feel appropriate. This may or may not include information on women with GDM. Recent qualitative work in London showed that new mothers knew little about the process of information sharing between midwife and health visitor and some expected their health visitors to know more than they did. Many saw the benefits of improved information sharing and would welcome this, so while some specific concerns were raised, the general consensus was a need for better communication pathways between midwives and health visitors [19]. This is clearly an essential step before a consistent approach to delivering opportunistic diabetes advice emerges.

Health visitors, as with other health professionals, juggle many competing demands. There therefore needs to be consideration as to how health visitors can maximise opportunities to fit in raising this additional health concern so that it is heeded and acted upon. This should take account of the other topics for discussion already contained within the HVP as well as ensuring the timing of the health intervention is raised before the potential seriousness of having GDM is erased from memory. Given that many women also consider a return to work, the suggested 6–8 month period home visit has potential as a time for delivering opportunistic advice around T2D risk.

Given that our research explored the views of HCPs, further research should be conducted to elicit the views of those women with GDM who would be in receipt of opportunistic advice, to explore whether such advice would be welcome and acceptable to them. While the findings here do echo those from a similar study in Denmark [20], there is still a need to repeat this study in larger samples of more diverse HCPs, in different health care context and settings.

## Conclusions

In summary, this study suggests that the frequency of routine visits with women during the Health Visiting Pathway (HVP) programme in Scotland provides potential opportunities to deliver opportunistic advice relating to behaviour change and diabetes risk to women who have had GDM. This is important because many women do not appreciate the potential consequences of GDM, or recognise their increased risk of T2D. Despite an already demanding schedule of providing advice and education to women, health visitors were confident that the HVP could be used to provide such interventions in the best interest of the mothers and their children. It is crucial, however, that the intervention does not undermine the confidence of the women, that it is evidence-based, that health visitors are prepared with appropriate training, and that the timing of the intervention is suitable. In addition, the identification of women with GDM should be streamlined within an effective recall system to allow health visitors to adequately plan and prepare to deliver advice that corresponds with routine consultations.

## Supplementary Information

**Additional file 1.** Interview Schedule for HCPs

## Data Availability

Research data can be made available to interested parties upon reasonable request to the corresponding author.

## Abbreviations

GDM: Gestational diabetes mellitus
T2D: Type 2 diabetes
HVP: Health visiting pathway
HCP: Health care professional
GP: General Practitioner
