# Supplementary material for: Postpartum opportunistic advice in primary care for women who have had gestational diabetes: a qualitative study of health care professionals’ views
Source: BMC Fam Pract. 2021 Oct 20;22:209. doi: 10.1186/s12875-021-01558-x (PMC8527649; doi:10.1186/s12875-021-01558-x)
Supplement: Supplementary file 1 — Additional file 1. Interview Schedule for HCPs [file 12875_2021_1558_MOESM1_ESM.doc]

**Interview Schedule for HCPs**

**Preamble**

I would like you to talk as descriptively as possible, expand on all your ideas (even if you think they are not related) and ask me to clarify anything you don’t understand. Your responses and your views will be kept anonymous and you will not be identifiable any research outputs.

**Main topics**

1. REACH

In your experience, how much information and advice do women who have had gestational diabetes receive after delivery, in terms of type 2 diabetes prevention?

Do you think these women would be receptive to such advice post delivery?

1. EFFECTIVENESS

What do you think about the intervention components for delivery to women who have had gestational diabetes? What do you think would be an optimal schedule for delivery of these components (in terms of HV appointments/smear visits)?

1. ADOPTION

Do you think women would agree to having this advice at a routine health care consultation?

1. IMPLEMENTATION

How would you feel about delivering the intervention components to women who have had gestational diabetes? Would you have time? When do you think would be the best time for this? **HVs/PNs**

Would you be happy about HVs/PNs delivering the intervention components to women in your practice who have had gestational diabetes? Would they have time? When do you think would be the best time for this? **GPs**

Do you think women who have had gestational diabetes should receive the intervention components in primary care? By whom? **Consultants**

1. MAINTENANCE

In your practice, how could call-up of women be operationalised? Is there any way of flagging up women who have had gestational diabetes? How could this work for health visitor appointments/ smear visits? **HVs/PNs/ GPs**
